# Could radiofrequency echographic multispectrometry (REMS) overcome the overestimation in BMD by dual-energy X-ray absorptiometry (DXA) at the lumbar spine?

**DOI:** 10.1186/s12891-022-05430-6

**Published:** 2022-05-19

**Authors:** Carla Caffarelli, Maria Dea Tomai Pitinca, Antonella Al Refaie, Michela De Vita, Simone Catapano, Stefano Gonnelli

**Affiliations:** Department of Medicine, Surgery and Neuroscience, University of Siena, Policlinico Le Scotte, Viale Bracci 2, 53100 Siena, Italy

**Keywords:** Osteoporosis, Osteoarthritis, Vertebral fractures, Radiofrequency echographic multi-spectrometry (REMS), Dual-energy X-ray absorptiometry (DXA)

## Abstract

**Background:**

Osteoarthritis (OA) and vertebral fractures at the lumbar spine lead to an overestimation of bone mineral density (BMD). Recently, a new approach for osteoporosis diagnosis, defined as radiofrequency echographic multi-spectrometry (REMS), represents an innovative diagnostic tool that seems to be able to investigate bone quality and provide an estimation of fracture risk independent of BMD.

The aim of this paper was to evaluate whether the use of REMS technology can favor the diagnosis of osteoporosis in subjects with an apparent increase in BMD.

**Methods:**

In a cohort of 159 postmenopausal (66.2 ± 11.6 yrs) women with overestimated BMD by DXA at the lumbar spine, we performed an echographic scan with the REMS technique.

**Results:**

The mean values of BMD at different skeletal sites obtained by the DXA and REMS techniques showed that the BMD T-scores by REMS were significantly lower than those obtained by the DXA technique both at the lumbar spine (*p* < 0.01) and at all femoral subregions (*p* < 0.05). In OA subjects, the percentage of women classified as “osteoporotic” on the basis of BMD by REMS was markedly higher with respect to those classified by DXA (35.1% vs 9.3%, respectively). Similarly, the REMS allows a greater number of fractured patients to be classified as osteoporotic than DXA (58.7% vs 23.3%, respectively).

**Conclusions:**

REMS technology by the analysis of native raw unfiltered ultrasound signals appears to be able to overcome the most common artifacts, such as OA and vertebral fracture of the lumbar spine, which affect the value of BMD by DXA.

## Background

Osteoarthritis (OA) and osteoporosis are two age-related degenerative diseases that are common in middle-aged and older women. Bone mineral density (BMD) by dual-energy X-ray absorptiometry (DXA) measurement is currently considered the gold standard for screening and monitoring bone status [1]. Not infrequently, artifacts and incidental findings may be observed that warrant recognition by the interpreting physician [2].

In fact, lumbar spine DXA is acquired as a 2D anteroposterior (AP) projection, and the resulting areal bone mineral density measurements can be confounded by structural abnormalities such as osteophytes and vertebral compression fractures [3]. Even though very severe calcifications of the aorta can lead to an overestimation of BMD, aortic calcifications have a minimal influence on BMD in the majority of cases [4]. Moreover, the treatment of vertebral fractures by vertebroplasty, especially when it involves two or more lumbar vertebrae, limits the possibility of obtaining an adequate evaluation of BMD by DXA [2].

Osteoarthritic changes are the most common source of artifacts encountered on DXA assessment, especially in adults and elderly patients. At the level of the lumbar spine, the manifestations of osteoarthritis are represented by send-plate osteophytosis, sclerosis, disk space narrowing, and facet joint arthropathy. Therefore, the presence of these structural abnormalities in a lumbar spine DXA scan can artificially increase the apparent BMD measurement [5].

The International Society for Clinical Densitometry (ISCD) Official Position, in order to overtake this trouble, has recommended excluding from the DXA analysis both the vertebrae with a greater than 1.0 T-score difference with respect to the adjacent vertebrae and those with important degenerative changes [6]. However, vertebral body exclusions lead to a small improvement in fracture prediction but also reduce the clinical value of DXA for monitoring [7]. Moreover, Blanck et al. reported that excluding vertebrae reduced precision due to the reduction of measured bone area [8].

For some years, radiofrequency echographic multi spectrometry (REMS) has been a promising new nonionizing technology available for evaluating bone status. The REMS technology is based on the analysis of native raw unfiltered ultrasound signals, the so-called radiofrequency (RF) ultrasound signals, obtained during an echographic scan of lumbar vertebrae and proximal femur [9]. The analysis of native unfiltered ultrasound signals permits the acquisition of maximum information regarding the characteristics of the evaluated tissues, which are normally filtered out during the conventional process of B-mode image reconstruction. The bone density is obtained through the comparison of the analysed signal spectra with previously derived reference spectral models for the considered pathological and normal conditions [9]. The diagnostic accuracy and precision of REMS compared to DXA have already been validated [10]. Recently, a large European multicenter study reported that REMS-measured T-score values were associated with the occurrence of previous osteoporotic fractures, even at a slightly higher degree than corresponding DXA T-score values [11]. Moreover, an Italian study by Adami reported that the REMS T-score is able to predict the occurrence of incident fragility fractures in women, representing a promising approach to enhance osteoporosis diagnosis in clinical routine [12].

This study aimed to evaluate whether the use of the REMS technique may improve the identification of osteoporosis status in subjects with an apparent increase in BMD due to the presence of artifacts.

## Patients and methods

### Patients

A cohort of 180 consecutive Caucasian women referred to the outpatient Clinic for Osteoporosis of the Department of Internal Medicine at the University Hospital of Siena (Italy) for an evaluation of BMD between May 2020 and December 2020 were enrolled in the study.

The inclusion criteria were as follows: age between 50 and 80 years, postmenopausal status, body mass index (BMI) between 18.5–39.9 kg/m^2^, presence of moderate/severe vertebral fractures or osteoarthritis at the lumbar spine, as confirmed by radiography taken in the previous six months. The patients previously treated with antiosteoporosis drugs, except calcium and vitamin D supplements, and those who were suffering illness (cancer, multiple myeloma, hyperparathyroidism, etc.) or were receiving therapies able to influence bone metabolism (glitazones, glucocorticoids, anticonvulsants, etc.), were excluded. Of the 180 enrolled patients, 21 were eliminated as a consequence of stringent inclusion/exclusion criteria (*N* = 15) and inadequate quality of REMS or DXA measurements (*N* = 6). In particular, the reasons for the inadequate quality of REMS or DXA measurements were inaccurate patient positioning (2 DXA and 2 REMS scans) and deviations from the acquisition procedure due to wrong or suboptimal settings of transducer focus (two REMS scans).

Of the 159 patients, 113 (mean age 63.2 ± 11.3 years) with radiological osteoarthritis at the lumbar spine and 46 (mean age 73.6 ± 18.5 years) with an atraumatic vertebral fracture at the lumbar spine were considered for analysis. All patients had normal serum creatinine levels and no major comorbidities impairing normal daily activity. In all, height and weight were measured in a standardized fashion, and body mass index (BMI) was calculated as weight in kilograms divided by the square of height in meters.

### Plain radiography

All radiological documentation was reviewed by two of the authors (C.C. and G.S.) with specific expertise. All lumbar radiographs were examined for the presence of any vertebral fracture according to Genant’s method [13]. Moreover, the presence of osteophytes was evaluated on lumbar spine X-rays according to the Kellgren/Lawrence grading system [14].

### Dual-energy X-ray absorptiometry

In all subjects, we measured BMD at the lumbar spine (LS-BMD), femoral neck (FN-BMD) and total hip (TH-BMD) using a dual-energy X-ray absorptiometry device (Discovery W, Hologic, Waltham, MA, USA). All DXA scans were performed according to standard clinical routine procedures. Osteoporosis and osteopenia were diagnosed according to the World Health Organization (WHO) definition: a T value lower than -2.5 was diagnosed as osteoporosis, and a T value less than -1.0 but higher than -2.5 was diagnosed as osteopenia; sex-matched Italian reference data were used for the calculation of the T-score [15].

### Radiofrequency echographic multispectrometry

Bone mineral density by REMS scans was performed employing a dedicated echographic device (EchoStation, Echolight Spa, Lecce, Italy) equipped with a convex transducer operating at the nominal frequency of 3.5 MHz.

The technical characteristics of the REMS device and the methods of carrying out the measurements have been described in detail in previous papers from our and other groups [9, 10, 12, 16, 17]. Briefly, the analysis of backscattered row signals allows us to obtain a spectral model for each subject that undergoes an advanced comparison with reference spectral models resulting in a BMD estimation and in the consequent diagnostic classification as normal, osteopenic or osteoporotic [10, 12, 16].

Informed written consent was obtained from all participants, and the study was approved by the Institutional Review Board of Siena University Hospital. All the data were anonymized before being used for the statistical analysis.

**Fig. 1:**
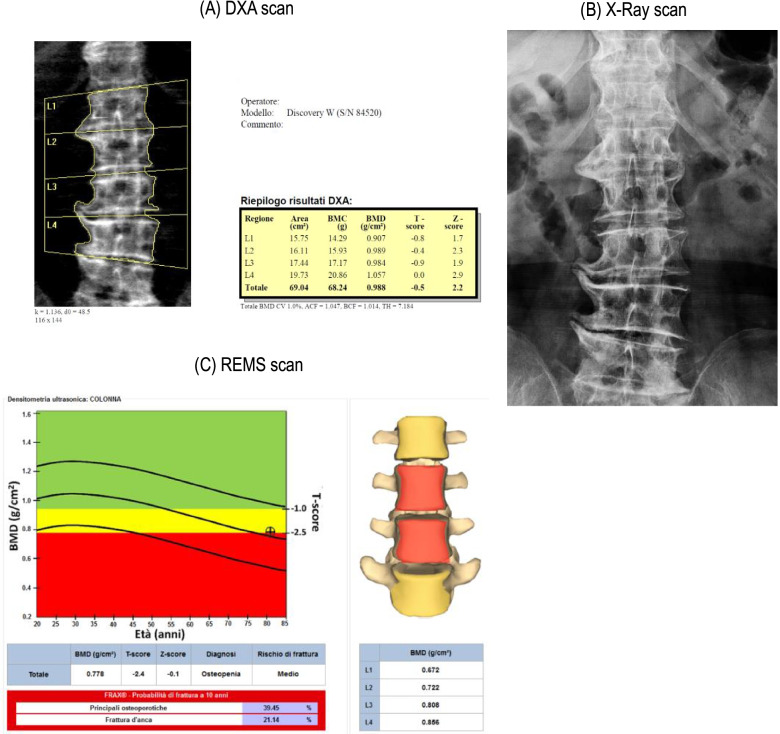
Schematic example of the subject’s lumbar spine assessment of BMD by DXA (**A**) and REMS (**C**) in a 69-year-old woman with severe osteoarthritis at X-ray scan (**B**)

Figure 1 shows a schematic example of the assessment of BMD by DXA (1A) and REMS (1C) in a 69-year-old woman with severe osteoarthritis on X-ray (1B).

## Statistical analysis

All values are expressed as the mean ± SD. The Kolmogorov–Smirnov test was used to verify the normality of the distribution of the outcome variables. Clinical data and initial values of the variables measured in the study groups were compared using Student’s t test and the Mann–Whitney U-test as appropriate. Categorical variables were compared by the chi-square test or Fisher’s exact test, as appropriate.

All tests were performed using the SPSS statistical package for Windows version 16.0 (SPSS Inc., Chicago).

## Results

The demographic and clinical characteristics of the study population are shown in Table 1. As expected, postmenopausal women with fragility fracture were older and shorter than those with osteoarthritis.

**Fig. 2:**
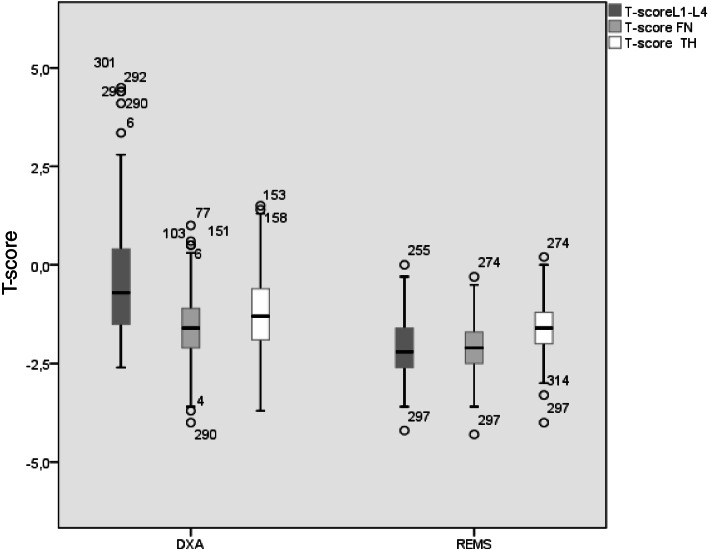
Values of BMD expressed as T-score at lumbar spine (LS), at femoral neck (FN) and at total hip (TH) by DXA and REMS technique in 159 postmenopausal women with fractures or osteoarthritis at lumbar spine

The mean T-score BMD values measured by the DXA and REMS techniques are shown in Fig. 2. As expected, the values of T-score BMD-LS by DXA were significantly higher (*p* < 0.05) with respect to BMD-LS by REMS; instead, at both femoral sites, the values of T-score by DXA were slightly higher only with respect to those assessed by REMS technique. Moreover, when considering DXA measurements, the T-score at LS was higher than those at both FN and TH.

**Fig. 3:**
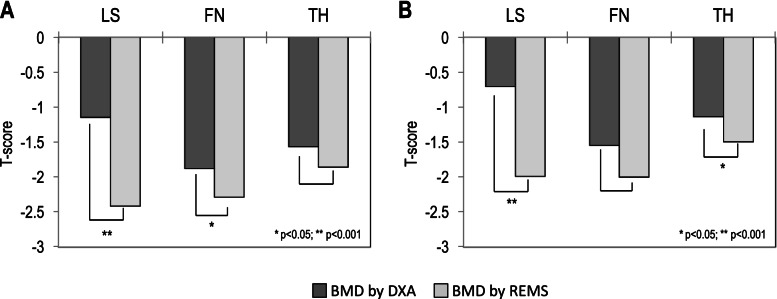
Values of BMD expressed as T-score at lumbar spine (LS), at femoral neck (FN) and at total hip (TH) by DXA and REMS technique in subject with fractures (**A**) and in subjects with osteoarthritis at lumbar spine (**B**)

Figure 3 shows the mean BMD values at different skeletal sites, expressed as T-scores, obtained by the DXA and REMS techniques in subjects with vertebral fractures (A) and in subjects with osteoarthritis at the lumbar spine (B). It is evident that the BMD-LS T-score by REMS​​ was significantly lower than that obtained by the DXA technique (*p* < 0.001). Additionally, at femoral sites, the T-score values by REMS ​​were lower than those obtained by DXA, but the difference was significant only for BMD-TH in women with osteoarthritis and for BMD-FN in women with fracture (*p* < 0.05).

**Fig. 4:**
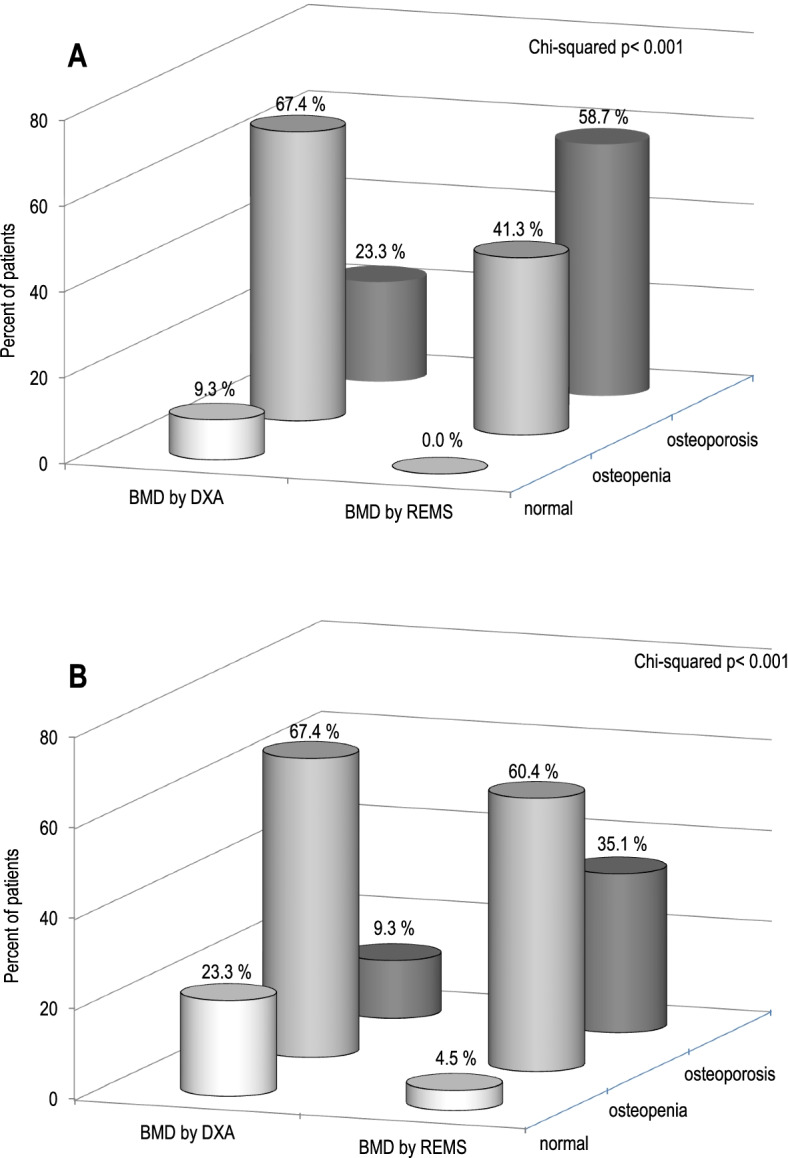
Percentage of fractured subjects classified as “osteoporotic”, “osteopenic” or “normal” on the basis of BMD T-score values obtained by DXA and REMS technique in subjects with fractures (**A**) and in subjects with osteoarthritis at the lumbar spine (**B**)

Figure 4 shows the percentage of women with fragility fractures at the lumbar spine (Fig. 4A) or osteoarthritis at the lumbar spine (Fig. 4B) classified as “osteoporotic”, “osteopenic” or “normal” on the basis of BMD T-score values obtained by the DXA and REMS techniques. It is evident that the REMS technique allows a greater number of patients with fracture to be classified as osteoporotic than DXA (58.7% vs 23.3%, respectively). In contrast, the percentage of women classified as osteopenic or normal by DXA was higher than that by REMS (67.4% and 41.3% vs 9.3% and 0.0%, respectively) (Fig. 4A). Similarly, the REMS technique allows a greater number of patients with osteoarthritis at the lumbar spine to be classified as osteoporotic than DXA (35.1% vs 9.3%, respectively). In contrast, the percentage of women with osteoarthritis at the lumbar spine classified as osteopenic or normal by DXA was higher than that by REMS (67.4% and 60.4% vs 23.3% and 4.5%, respectively) (Fig. 4B).

## Discussion

To the best of our knowledge, this study represents the first report on the usefulness of REMS to enhance the diagnosis of osteoporosis when lumbar spine BMD by DXA is impaired by artifacts due the presence of fragility fractures or osteoarthritis changes. Dual energy X-ray absorptiometry (DXA) represents the “gold” standard for the measurement of bone mineral density (BMD) and is an integral part of the fracture risk assessment process [18, 19]. Nevertheless, DXA does have limitations; in fact, only quantitative information is produced from DXA two-dimensional scan images (i.e. areal BMD), and no qualitative three-dimensional information relating to bone structure can be obtained [20]. Moreover, the diagnostic accuracy of BMD measurement by DXA can be markedly reduced by the presence of artifacts. In our overall population, the mean BMD-LS T-score by DXA was in the normal range, whereas the mean BMD-LS T-score by REMS was in the osteopenic range. Therefore, in this type of patient, the correct prediction of fragility fracture risk remains a challenge.

In particular, the accuracy of the BMD measured by DXA to predict fracture is misleading in subjects who have presented with an atraumatic vertebral fracture at the lumbar spine and who have radiological osteoarthritis of the lumbar spine [21]. Several studies have reported that osteoarthritic spondylosis is the most common cause of artefactual increases in BMD values due to abnormally dense bone at osteophytes, facet joint sclerosis and vertebral margins forming vertebral end-plate sclerosis. The artifactual elevation of BMD is more marked in lower lumbar vertebrae, providing recognized evidence of progressive osteoarthritic alterations seen in sequential descending vertebrae, which correlates with increasing BMD values caudally down the lumbar spine [22].

For many years, osteoporosis and osteoarthritis, two common age-related diseases responsible for great morbidity and functional impairment, have been considered independent. Recently, it has been reported that osteoporosis and osteoarthritis are related by complex and intriguing relationships. Several cross-sectional and longitudinal studies have reported the coexistence of osteoarthritis and osteoporosis. Moreover, these studies have demonstrated an unexpectedly increased risk of fragility vertebral fractures in subjects with spondyloarthritis [23].

REMS technology by the analysis of natural raw unfiltered ultrasound signals appears to be able to recognize and automatically remove the raw signal of osteophytes, calcifications and other possible causes of artifacts, thus permitting a correct definition of osteoporosis and consequently a better assessment of fragility fracture risk [16, 17, 24]. However, the lack of published data on the accuracy of REMS in subjects with BMI > 40 kg^/m2^ may represent a limit to the use of REMS in very severely obese patients.

The ability of REMS and DXA T-score to identify subjects at risk of fragility fracture incidence has been investigated in a recent prospective Italian study by Adami et al. [12]. This study reported that REMS was better with respect to DXA in order to identify individuals at risk of fragility fractures, as demonstrated by the higher AUCs of REMS T-score values than those DXA obtained for the discrimination between cases with and without incident fragility fracture. The identification of a higher number of patients with osteoporosis by REMS with respect to DXA might be correlated with a possible higher increased ability of REMS to diagnose osteoporosis and assess fragility fracture risk [25, 26].

At present, there is a growing interest in identifying new easy-to-use and reliable techniques that can improve our ability to assess bone status and fracture risk. In particular, REMS techniques may represent a promising tool to assess some qualitative bone properties; moreover, REMS presents some advantages over DXA, such as the absence of ionizing radiation, portability and low cost [11, 17, 27].

In this contest, the Italian ministerial, inter-societal guidelines for the “Diagnosis, risk stratification and continuity of care for Fragility Fractures” issued in October 2021 recognized the REMS ultrasound examination as a diagnostic technology that can facilitate the patient's care pathway [28].

This study presents some limitations. First, the cross-sectional nature of the study does not allow the establishment of any causal relationships between the parameters. Second, the number of patients enrolled in the present study is relatively small. Third, there was a lack of any third reference technique, such as QCT, to confirm the truthfulness of REMS-derived BMD values. Finally, women with severe obesity were excluded.

## Conclusions

This study found that REMS technology may represent a promising approach to enhance osteoporosis diagnosis in subjects with an apparent increase in BMD due to the presence of vertebral fractures or osteoarthritis at the lumbar spine. Further studies are warranted to confirm these preliminary data and to establish the usefulness of REMS for a better fracture risk evaluation in patients with overestimated BMD by DXA at the lumbar spine.

**Table 1:**
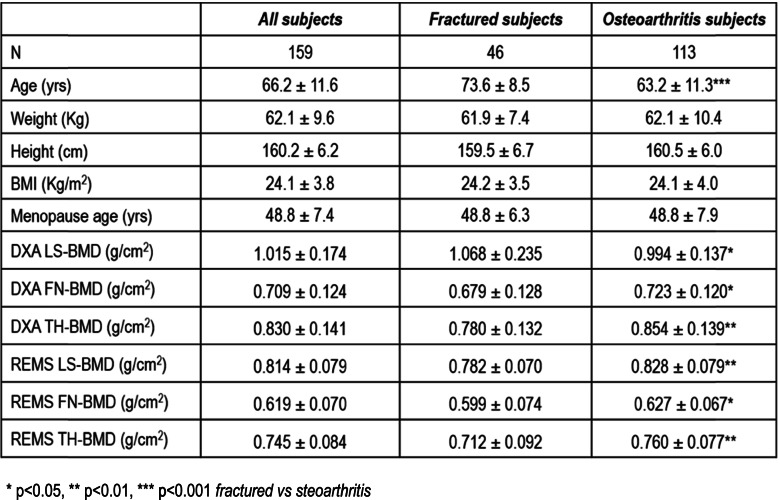
Demographic and clinical characteristics of the study population

## Data Availability

The datasets generated and/or analyzed during the current study are not publicly available due to limitations of ethical approval involving the patient data and anonymity but are available from the corresponding author on reasonable request.

## Abbreviations

REMS: Radiofrequency Echographic Multispectrometry
BMD: Bone mineral density
DXA: Dual-energy X-ray absorptiometry
BMI: Body mass index
LS: Lumbar spine
FN: Femoral neck
TH: Total hip
